# Therapeutic effects of *Ghrita Manda Ashchyotana* on tear film stability, corneal integrity, and IL-6 expression in a benzalkonium chloride-induced murine dry eye model

**DOI:** 10.1016/j.jaim.2026.101354

**Published:** 2026-05-27

**Authors:** Ramya Shruthi Janardhanan, Sivabalaji Kundrinmani, Ashwini Belludi Nagaraj

**Affiliations:** Department of Shalakya Tantra, Amrita School of Ayurveda, Amrita Vishwa Vidyapeetham, Kollam, Kerala, India

**Keywords:** *Ghrita manda*, *Dry eye*, *IL-6*

## Abstract

**Background:**

Dry Eye (DE) is a condition marked by tear film instability and chronic inflammation, leading to visual impairment. Conventional artificial tears often contain preservatives that exacerbate DE and fail to address underlying tissue damage. In *Ayurveda,* DE symptoms corelates with *Sushkakshipaka*, managed by normalizing *vata pitta* derangement. This study evaluates the mechanism of *Ghrita MandaAshchyotana* in a Benzalkonium Chloride (BAC)-induced murine dry eye model.

**Methodology:**

12 male Swiss albino mice (24 eyes) were divided into: saline-treated controls (3 mice), and dry eye-induced mice treated with either *Ghrita Manda* (Trial), preservative-free eye drops (Positive control), or left untreated (Negative control). Assessments included Tear Film Break-Up Time (TFBUT), fluorescein scores, corneal Hematoxylin and Eosin (H&E) staining, and IL-6 expression after 14 days.

**Results:**

*Ghrita Manda* significantly improved TFBUT and reduced fluorescein scores compared to the negative control (p < 0.001). Corneal integrity was preserved without hyperplasia in the trial group. A notable reduction in IL-6 expression was observed compared to the positive control.

**Conclusion:**

*Ghrita Manda* demonstrates potential as an effective DE therapy, offering lubrication, corneal protection, and anti-inflammatory benefits, aligning with *Ayurvedic* principles for managing *vata pitta* derangements of the ocular surface.

## 1 Introduction

The Tear Film and Ocular Surface Society (TFOS), Dry Eye Workshop II (TFOS DEWS II), defines Dry Eye (DE) as *‘‘a multifactorial disease of the ocular surface characterized by a loss of homeostasis of the tear film, and accompanied by ocular symptoms, in which tear film instability and hyperosmolarity, ocular surface inflammation and damage, and neurosensory abnormalities play etiological roles”* [1]. Between 5 and 50% is the usual incidence of DE [2]. DE poses a tremendous financial burden on society because of its detrimental impacts on vision, quality of life, and workplace productivity [3,4]. Aqueous deficient dry eye (ADDE) and Evaporative dry eye (EDE) are the clinical classification of the condition. However, mixed form of Dry Eye involving both evaporative and aqueous component exists around in 80 % of the cases [5]. The pathophysiology of the dry eye involves ocular epithelial cell damage results from tear instability and hyperosmolarity, leading to cell stress signalling pathways and produces innate inflammatory molecules like chemokines, cytokines, and matrix metalloproteinases (MMPs). It also excites the ocular nerve endings, which can result in ocular discomfort, an increased blink rate, and possibly a reflex tear secretion [6]. Lubrication, ocular epithelial protection and the management of inflammation forms the triad in the treatment. Of these, artificial tears as lubricants are prescribed as a main stay of the treatment which can be in the form of demulcent or emollient agents which are primarily intended to protect, lubricate and replenish the tear component of the ocular surface. The dosage of these lubricants can vary from 3 to 4 times a day depending on the severity [7].

Multi-dose lubricants are often laden with preservatives and the most widely used is Benzalkonium chloride (BAC) of concentration 0.004 % and 0.2% to maintain the sterility of the ophthalmic formulation [8]. In vivo and In vitro models show cytotoxic signs upon the instillation of BAC in the concentrations used as ophthalmic preservatives [9]. Short term exposure (∼7 days) of the ocular surface tissues has been documented with apoptosis of corneal epithelial cells, loss of conjunctival goblet cells, delayed corneal wound healing, lymphocyte infiltration and increase levels of inflammatory markers in the ocular tissues [10]. Incidence of OSD in patients using long term preservative containing eye drops as a part of glaucoma treatment is shown to be 30% −70% [11,12] Usage of BAC preserved eye drops in pre-existing OSD like dry eye is found to worsen the dry eye cycle. Hence for long standing dry eye conditions, preservative-free single vial drops are mostly recommended [13]. Limitations of the preservative-free drops include frequent contamination of the eye drop leading to microbial keratitis, patient-related usage difficulties, reduced shelf-life and are expensive [14,15].

In Ayurveda, the term ocular surface disease can be co related to the symptoms of *sushkakshipaka* explained in the context of *sarvakshi roga* [16]*. Madhukosha* explains *sushkakshipaka* as *paka* (inflammation) of *netra* which develops as a result of *sushkata (dryness)* leading to *netra upahata (damage to eye)* [17]*.* The *dosha* predominance of *sushkakshipaka* ranges from *vata, vata pitta* and *vata rakta* by *Sushruta, Vagbhata and Sharangadhara* respectively [[18], [19], [20], [21]]. *Ashru* (tear film) is the byproduct of *rasa, meda, asthi* -*majja dhatu* [[22], [23]] and the disease manifestation occurs when there is derangement of the dosha in these *dhatu* of eyes. Management of *sushkakshipaka* involves *sarpi pana* (internal oleation)*, anu taila nasya (instillation of anu taila through nasal route), kshirasaindhava seka* (ocular therapy by streaming of medicated milk)*, anjana (collyrium) with mahaushadhi yoga, keshamashi yoga, tarpana* (therapeutic retention of medicated ghee or scum of ghee over the eyes), *jivaniyagana, basti* (therapeutic unctuous enema) and utilisation of *Ghrita Manda* [[24], [25], [26]]*. Ghrita Manda* is the supernatant, clear, unctuous layer of the melted *Ghrita* (ghee) which is devoid of the *ghanabhaga* (residue). This is referred to as the scum of ghee. It is indicated in the *shula* (painful conditions) of *yoni* (vagina), *srotra* (ear), *akshi* (eye), *shiras* (head). It can be incorporated in the therapies like *basti* (enema), *nasya* (medication through nasal route), *akshipoorana* [*Ashchyotana* (eye drop)/ *tarpana* (therapeutic retention of medicated ghee or scum of ghee over the eyes)] and useful in the derangement of *vatapitta dosha* [27,28]. In Ayurvedic texts it is explained that the *Ghrita Manda* can be used in the form of eye drops.

Although the effectiveness of *Ayurvedic* treatment protocols in *sushkakshipaka* has been established in clinical trials [22,29,30], corneal histopathology and inflammatory marker studies are not available to interpret its mechanism. Hence this study aims to demonstrate the effect of *Ghrita Manda* *Ashchyotana* in Benzalkonium Chloride (BAC) induced murine Dry Eye model.

## 2 Materials and methods

### *2.1* *Ghrita Manda* preparation

Classical *Ayurvedic* method of preparation of *Ghrita* (ghee) was adopted for obtaining the study drug *Ghrita Manda* (Scum of ghee). *Go kshira* (Cow's milk) of about 90 L was collected from the grass-fed cow (*Bos indicus*) and was boiled at 120 °C and was allowed to cool in the room temperature. 10% of curd which was previously prepared was added to the milk and was allowed to ferment overnight to obtain *dadhi* (curd). The next day, the curd was churned to obtain *navanita* (butter) and the *takra* (buttermilk) was strained out. Approximately 1.550 kg of butter was obtained. The butter was then transferred into a clean, wide-mouthed vessel and was heated over a medium, steady flame (∼65 °C) until all the moisture was evaporated and the characteristic aroma of ghee was obtained. The *Ghrita* (ghee) was transferred in to a glass jar and left undisturbed for few hours until the solid residue settles in the bottom of the jar. The clear supernatant liquid portion is called *Ghrita Manda* (scum of the ghee) of approximately 500 gm was obtained and stored in an air-tight glass jar.

### 2.2 Quality control analysis of *Ghrita Manda*

Physio chemical analysis of *Ghrita Manda* was done in the quality control lab, department of *Rasa Shastra & Bhaishajya Kalpana*, Amrita School of Ayurveda, Kollam, Kerala.

### 2.3 Sterility test of *Ghrita Manda*

Sterility test of the sample was done at Amrita Centre for Advanced Research in Ayurveda.

(ACARA) with Test Requisition No: ACARA052/24-25.

**Materials Required:** Nutrient Agar (NA) (Merck, Germany), Saboraud Dextrose Agar (SDA) (Merck, Germany, Autoclaved peptone water, sterile petri plates, Micropipettes, Autoclaved Pipette tips, Petri plates, Conical Flask, Biosafety cabinet II, Spirit, Bunsen burner, BOD incubator, Colony counter, Discard beaker.

**Procedure:** Clean the place of work inside Biosafety cabinet using spirit / 70% ethanol, keep materials required for the study inside the cabinet and switch on UV for 20 min. Prepare 100 ml of both media and peptone water, autoclave. Take 1g of sample and mix in 9 ml of sterile peptone water to make 10-1 dilution in the sterile environment. Pour the medium into sterile Petri dishes, add 100 μl of diluted sample into the Petri dish containing the media. Mark the name of the sample correctly on the Petri dish. Gently rotate the plate in circular motion to achieve uniform distribution of the sample and allow the media to solidify. One plate is taken without any sample as negative control. Incubate NA plates at 37C for 24 h and SDA plates at 35 °C for48 h. Examine plates under test after incubation.

$$\begin{array}{r} Concentration\;of\;microbes\;\left(CFU/ml\right)=Number\;of\;colony\;forming\;units\;\left(CFU\right)\;X\;dilution\\ \frac{factor\;X\;conversion\;factor}{\text{Volume}\;of\;\text{diluted}\;\text{media}\;\text{with}\;\text{bacteria}\;\text{plate}} \end{array}$$

#### 2.3.1 Gram staining of the sample

##### 2.3.1.1 Preparation of a slide smear

An inoculation loop is used to transfer a drop of suspended culture to the microscope slide.

If a Petri dish or a slant culture tube has the colony, a drop or a few loopfuls of water is added to facilitate a minimal amount of colony transfer to the examination slide. A minimal amount of culture is required. If culture can be detected visually on an inoculation loop, it indicates the collection of too much culture. Culture is spread with an inoculation loop to an even thin film over a circle of 15 mm in diameter. A typical slide can contain up to 4 small smears if examining more than one culture. The slide can be either air-dried or dried with the help of heat over a gentle flame. The slide should be moved circularly over the flame to prevent overheating or forming of ring patterns in the slide. The heat helps the cell adhesion to the glass slide and prevents the significant loss of culture during rinsing.

**Gram staining:** Crystal violet stain is added over the fixed culture. After 10 to 60 s, the stain is poured off, and the excess stain is rinsed with water. The goal is to wash off the stain without losing the fixed culture. Iodine solution is used to cover the smear for 10 to 60 s. This step is known as "fixing the dye." The iodine solution is poured off, and the slide is rinsed with running water. Excess water from the surface is shaken off. A few drops of decolorizer are added to the slide. Decolorizers are often the mixed solvents of ethanol and acetone. This step is known as "solvent treatment." The slide is rinsed with water for 5 s. To prevent excess decolorization in the gram-positive cells, stop adding decolorizer as soon as the solvent is not colored as it flows over the slide. The smear is counterstained with basic fuchsin solution for 40 to 60 s. The fuchsin solution is washed off with water, and excess water is blotted with the bibulous paper. The slide can also be air-dried after shaking off excess water. Slide is observed under oil immersion microscope.

### 2.4 GC-MS of *Ghrita Manda*

The study drug's GC-MS analysis was carried out at Chennai's Greens Med Labs. The test material was subjected to GC-MS analysis using the Clarus 500 PerkinElmer Auto system XL. A PerkinElmer Turbo mass 5.2 spectrometer with an Elite - 5MS (5% Diphenyl/95% Dimethyl Polysiloxane) and a 30 m × 0.25 μm IIF capillary column is linked to a turbo mass gold gas chromatograph. The instrument's starting temperature was set at 110 °C, and it remained there for 2 min. Following this, the oven's temperature was increased by 5 °C every minute to 280 °C. 70 eV was the ionization voltage. In split mode, the test sample was injected 10:1.

GC-MS Instrument used was Agilent GC 7890A/ MS5975C, Capillary column: Agilent DB5MS with column length: 30 m/ 0.25 mm internal dia/ 0.25-μm film thickness. Using NIST mass spectral library the relative concentration of the phytochemical compounds in the test sample was expressed as a percentage based on the peak arm produced in the chromatogram.

### 2.5 BAC induced murine dry eye model

Twelve male Swiss Albino mice of 8 to 10 weeks of approximately 25 - 30 g were procured from the Animal Experiment center, SDM Centre for Research in Ayurveda and Allied Sciences, Udupi and sheltered under controlled laboratory conditions with room temperature 25 °C ± 1 °C, relative humidity 60% ± 10%, and alternating 12 h light-dark cycles. (8 a.m. to 8 p.m.). The study protocol was approved by the Institutional Animal Ethics Committee (IAEC), of SDM Centre for Research in Ayurveda and Allied Sciences, Udupi (SDMCRA/IAEC/AS.S.17) and the Committee for the Purpose of Control and Supervision of studies on Animals (CPCSEA) criteria were followed in conducting the studies. Diseased mice and mice which are used for other experiments were excluded from the study.

### 2.6 Experimental procedure

After the assignment of the mice into groups, the negative control group (n = 6 eyes) was left untreated for the baseline comparison. The trial group (n = 6 eyes) received 1220 μl of *Ghrita Manda* topically and the positive control group (n = 6 eyes) received Systane Ultra® PF lubricant eye drops and the control group (n = 6 eyes) received equal amount of saline. The trial, positive control and the control groups received the doses 4 times a day (8:00AM,12:00PM, 4:00 p.m., 8:00PM) for 14 days.

On the day 1 and day 14 of the trial, the clinical evaluation involving the Tear Film Break Up Time (TFBUT) and the fluorescein stain were conducted by an Ayurvedic ophthalmologist in a blinded manner. On the day 15, the mice were euthanized and the corneal tissues were carefully harvested and the tissues were sent for Hematoxylin & Eosin Staining (n = 2 eyes/ group) and Western blot analysis.

### 2.7 Fluroscein staining and tear film break up time (TFBUT)

Diethyl ether (SISCO CHEM) was used as an inhalant anesthetize the animals. To measure the TFBUT, 1 μL of 0.1% liquid sodium fluorescein mixed with a topical anesthetic (Paracaine 0.1% eye drop) was administered to the mice's conjunctival sac. The eye was held open and examined using a slit lamp biomicroscope under a cobalt blue filter after the lids were manually closed three or four times. The TFBUT (in seconds) was recorded. The beginning of dry spots is identified in Stop Watch. Corneal epithelial damage was graded using the staining score recommended in the DEWS II Diagnostic Methodology report, and the assessment methodology was adopted from a previously conducted in vivo study [31]. Four quadrants of the cornea were separated and assessed independently. The final grade was calculated by adding the scores from these four regions. For scoring purposes, grading was carried out as:

No staining – 0.

Mild punctate staining with fewer than 30 spots – 1.

Punctate staining with more than 30 spots but not diffuse – 2.

Severe diffuse staining without a positive plaque – 3.

Presence of a positive fluorescein plaque – 4.

### 2.8 Hematoxylin and EOSIN (H&E) staining

For 48 h, the tissue was preserved in 10% formalin. To move the tissue from the last wax bath to a mold that was filled with melted paraffin wax, the tissue was blocked or embedded. Thin sections of tissues block of 4 μm were cut with the help of microtome. The tissue sections are floated in water bath of temperature 50°- 52° and then taken in microscopic slides. Coverslip was placed in the slide and the prepared slides were seen under the LX-500 LED trinocular Research microscope (Labomed) and images were taken with MiaCam.

Image AR Pro software is attached to a CMOS AR 6pro microscope camera.

### 2.9 Western blot analysis

The total proteins from the tissues were extracted using RIPA buffer containing Phenyl Methane Sulphonyl Fluoride (PMSF). 20 μg of protein sample was loaded along with 15 μL of loading dye. The dilution rate of both Primary and secondary antibodies was 1:5000. The molecular weight of target protein is 22 kDa.The tissue samples were homogenized using a sterile surgical blade and sonicated for 30 s at 40 amp using Oscar Ultrasonics sonicator system. The sonicated samples were centrifuged at 10,000 rpm for 10 min at 4 °C to eliminate debris. The Protein estimation from the lysate was performed using Bicinchoninic acid assay (BCA) method. The protein was quantified using Biotek Epoch Microplate Spectrophotometer (Agilent Technologies). The protein samples were loaded on two 10 % SDS-PAGE gels and separated and further being transferred to nitrocellulose blotting membrane (Himedia). The membranes were then blocked with 1% BSA in Tris-buffered saline with Tween® 20 detergent (TBST) at room temperature for 60 min, incubated with IL-6 and β-actin primary antibodies separately on the respective transferred blots at 4 °C overnight. After the incubation the blots were washed using TBST, and incubated with appropriate horseradish peroxidase-conjugated secondary antibodies for 60 min and washed. Clarity Western ECL Substrate (Bio Rad) was used to detect the bands, GBox Gel Documentation unit (Chemi XX9, Syngene) was used to visualize. The comparative intensity of the bands was analyzed with the target protein IL-6 expression versus the house keeping gene β-actin using Gene tools software version Gene Tool Version 4.3.9.0 (Syngene). The height of the absorption peak was correlated with the intensity of the band provided by the Gene tools software. The values for IL-6 were normalized with the values of β-actin and are presented in the form of bar graph which represents IL-6 expression for the respective samples.

### 2.10 Statistical analysis

Statistical analysis was done with SPSS software version 30.0.0.

One-way ANOVA Test was employed to analyze the clinical observations between the groups at different time point of the trial followed by the Turkey multiple T-test to compare the difference between the groups.

## 3 Results

### 3.1 Action of *Ghrita MandaAshchyotana* on tear film instability

Evaluation of TFBUT in the mice (n = 6 eyes/group) before and after the experimentation was performed and the results are depicted in Fig. 1A.

**Fig. 1A:**
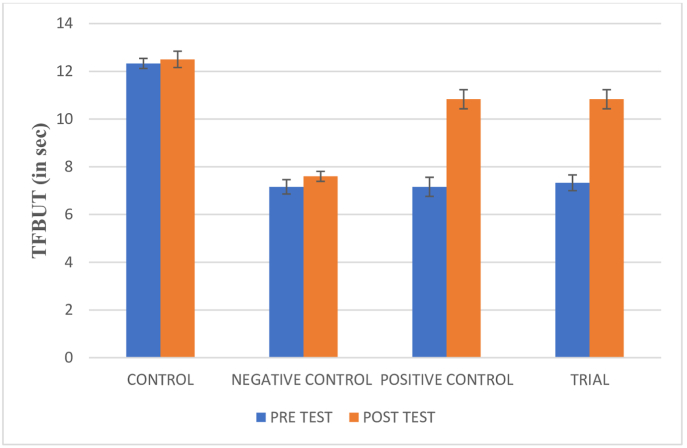
Action of *Ghrita MandaAshchyotana* on tear film instability.

### 3.2 Action of *Ghrita MandaAshchyotana* in fluorescein score

The fluorescein score was assessed before and after the experimentation and the results are depicted in Fig. 1B, Fig. 1CB and C.

**Fig. 1B:**
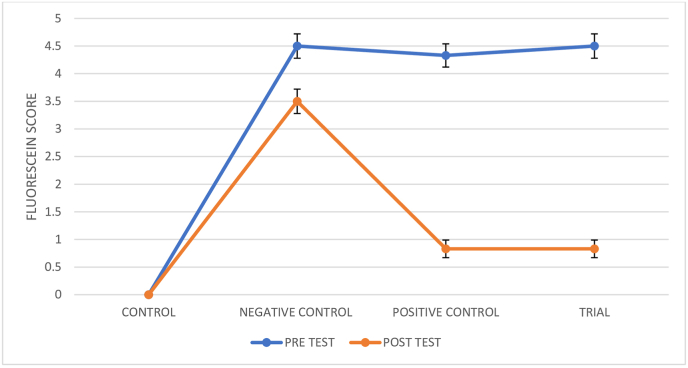
Action of *Ghrita MandaAshchyotana* in fluorescein score.

**Fig. 1C:**
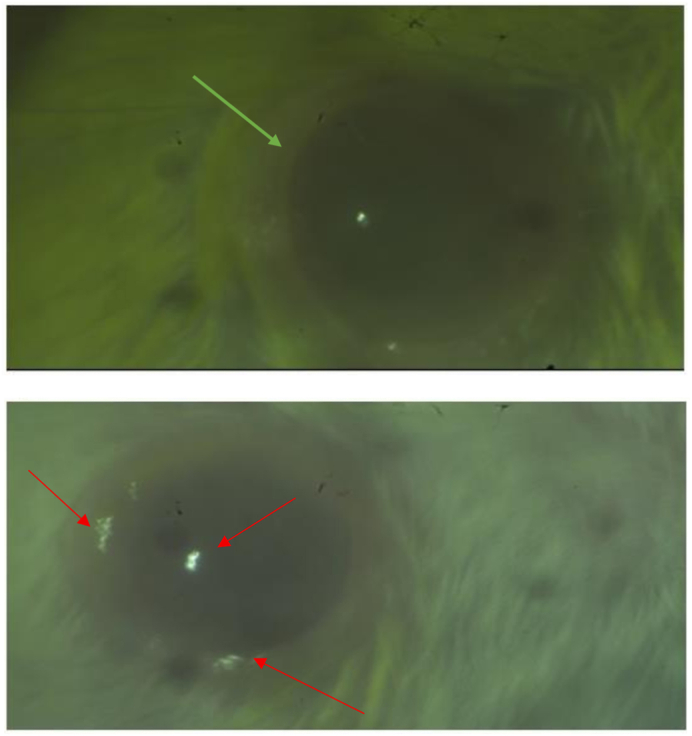
Fluorescein score photographs.

### 3.3 Action of *Ghrita MandaAshchyotana* on corneal epithelial damage

Corneal assessment was done to analyze the corneal damage and to estimate the extent of its recovery post treatment. Results of which are shown in Fig. 3A, Fig. 3B (Red arrow-inflammatory infiltrate; Blue arrow-epithelial hyperplasia; Yellow arrow-blood vessels).

#### 3.3.1 Control sample 1

**Fig. 2:**
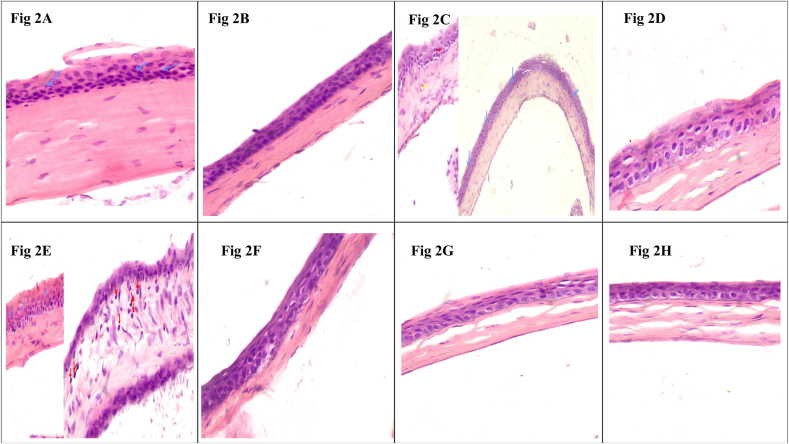
A: Control Cornea, B: Control Cornea, C: Negative Control Cornea, D: Negative control Cornea, E: Positive Control Cornea, F: Positive control Cornea, G: Trial Cornea, H: Trial Cornea.

The corneal epithelium is 5-6 layers thickness. The slide shows increased epithelial thickness (hyperplasia) compared with negative control. The cells show hyperchromatic nuclei. No inflammatory cells and neovascularization. (Shown in Fig. 2A- Control Cornea).

#### 3.3.2 Control sample 2

The corneal epithelium is 3-4 layers thickness. Histological changes like inflammatory infiltration, new blood vessel formation (neovascularization) is not seen in slide. The collagen stroma is organized with few keratinocytes in the stroma. (Shown in Fig. 2B- Control Cornea).

#### 3.3.3 Negative control sample 1

The cells show increased epithelial cell layers (hyperplasia), mild lymphocytic infiltrate and new blood vessels (neovascularization). The collagen stroma is organized with few keratinocytes in the stroma. (Shown in Fig. 2C- Negative Control Cornea).

#### 3.3.4 Negative control sample 2

The corneal epithelium is 3-4 layers thickness. Histological changes like inflammatory infiltration, new blood vessel formation (neovascularization) is not seen in the slide. The collagen stroma is organized with few keratinocytes in the stroma. (Shown in Fig. 2D - Negative Control Cornea).

#### 3.3.5 Positive control sample 1

The corneal epithelium is 5-6 layers thickness. The slide shows increased epithelial cell layers (hyperplasia) compared with negative control. One area of stroma shows mild acute (neutrophils) and chronic (lymphocytes). Disorganization and separation of collagen lamella seen in one area. Epithelium shows cells undergoing mitosis. (Shown in Fig. 2E- Positive Control Cornea).

#### 3.3.6 Positive control sample 2

The corneal epithelium is 3-4 layers thickness. Histological changes like inflammatory infiltration, new blood vessel formation (neovascularization) is not seen in slide. The collagen stroma is organized with few keratinocytes in the stroma. (Shown in Fig. 2F- Positive Control Cornea).

#### 3.3.7 Trial sample 1

The corneal epithelium is 3-4 layers thickness. Histological changes like inflammatory infiltration, new blood vessel formation (neovascularization) is not seen in slide. The collagen stroma is organized with few keratinocytes in the stroma. Compared with positive control, there is no histological changes. (Shown in Fig. 2G- Trial Cornea).

#### 3.3.8 Trial sample 2

The corneal epithelium is 3-4 layers thickness. Histological changes like inflammatory infiltration, new blood vessel formation (neovascularization) is not seen in slide. The collagen stroma is organised with few keratinocytes in the stroma. Compared with positive control and control 2, there is no histological changes. (Shown in Fig. 2H- Trial Cornea).

### 3.4 Action of *Ghrita MandaAshchyotana* on inflammatory marker (IL-6)

Western blot analysis was used to study the response of the inflammation in Dry Eye with reference to Interleukin-6 in mice cornea. Control (2.99 ± 0.487), Trial (3.63 ± 0.023), Positive control (3.85 ± 1.56) and Negative control (4.75 ± 1.73) groups showed gradual increase in expression of IL-6. Control group showed lower and Negative control group showed higher level of expression of IL-6 compared to β-actin respectively. Here, Tl and T8- Negative Control; T2 and T6- Positive control; T3 and T4- Control; T5 and T7 - Trial. (Shown in Fig. 3A, Fig. 3B, Fig. 4).

**Fig. 3A:**
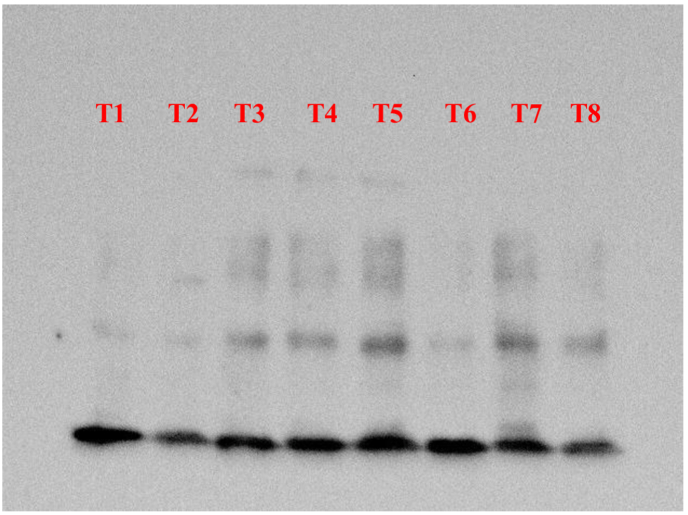
Western Blot analysis of target protein (IL-6).

**Fig. 3B:**
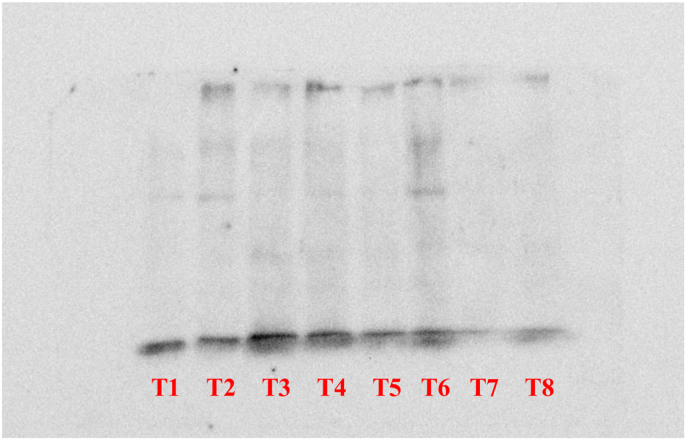
Western Blotting of housekeeping protein (β-Actin).

**Fig. 4:**
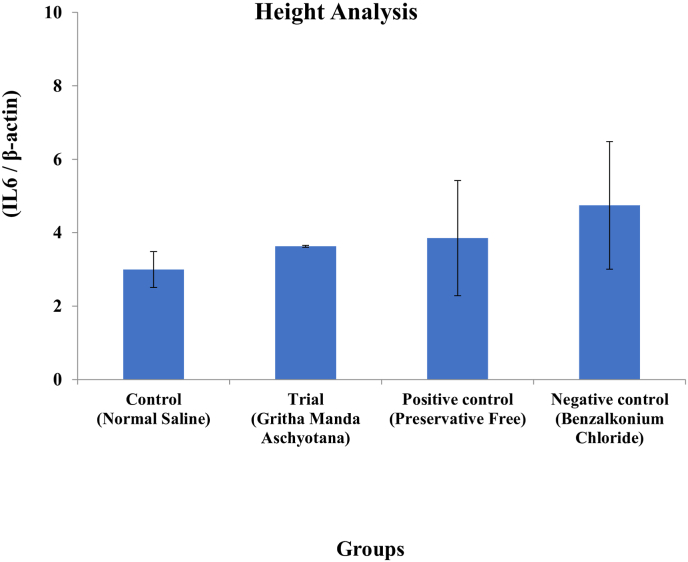
IL-6 Height Analysis using β-actin.

### 3.5 GC-MS analysis of *Ghrita Manda*

The GC-MS analysis of *Ghrita Manda* showed the presence of fatty acids and their derivatives.

(Shown in Fig. 5).

**Fig. 5:**
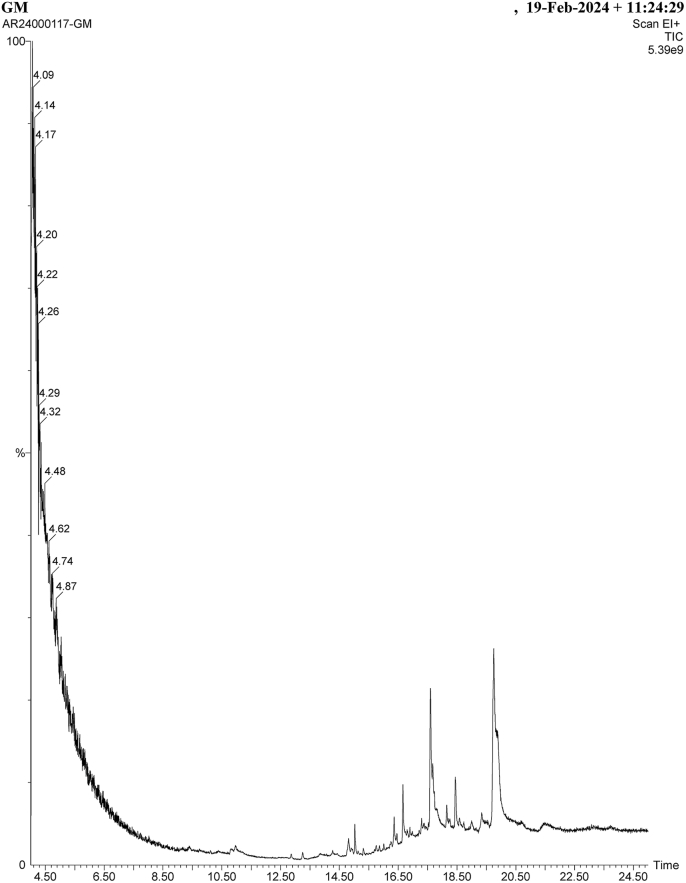
Chromatogram of *Ghrita Manda* (Scum of ghee).

#### 3.5.1 Among the identified compounds, the following were present in high concentrations

N-hexadecanoic acid (44.9%), Glycidyl palmitoleate (38.6%), Glycidyl palmitate (25.5%), 9,12-Octadecadienoic acid (18.7%), oleic acid (13.2 %).

The top five compounds with retention time, molecular formula, percentage abundance, pharmacological relevance are provided. (Shown in Table 1).

**Table 1:** The top five components identified by GC-MS and their properties.

Compound	Chemical formula	Retention Time (RT)	Percentage abundance	Pharmacological relevance
n-Hexadecanoic acid	C16H32O2	RT-16.6	44.9%	Antioxidant, Lubricant Anti- inflammatory
Glycidyl palmitoleate	C19H34O3	RT-18.4	22.5%	Anti-inflammatory Wound healing Restores tear secretion.
		RT-19.7	38.6%;	
Glycidyl palmitate	C19H36O3	RT-18.4	25.5%;	The component that forms lysophosphatidic acid in inhibiting apoptosis.
9,12- Octadecadienoic acid	C18H32O2	RT 17.6	18.7%	Anti-inflammatory Anti-oxidant Improves tear production
Oleic Acid	C18H34O2	RT-16.6	13.2%	Anti-inflammatory Immunomodulator Lubricant and emollient

### 3.6 Quality control analysis of *Ghrita Manda*

Quality control analysis was conducted after five months of *Ghrita Manda* storage to evaluate the stability of the product (Shown in Table 2).

**Table 2:** Quality Control report of *Ghrita Manda*.

S. No	Physico-Chemical Parameter	Result
1	Loss on Drying at 110 °C	0.3 % w/w
2	Specific Gravity at 28 °C	0.912
3	Refractive Index at 28 °C	1.4580
4	Peroxide Value	0.39 meq/kg
5	Acid Value	0.93
6	Saponification Value	166.5
7	Iodine Value	33.05
8	Viscosity at 28 °C	48–49 cp
9	Ash Value	Nil
10	pH	≈5
11	Ester Value	165.57

### 3.7 Sterility report of *Ghrita Manda*

**Fig. 6:**
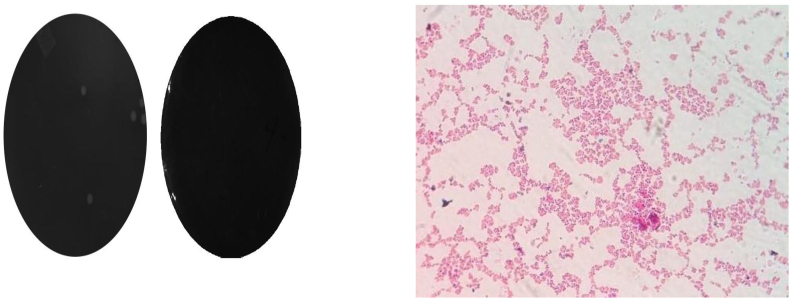
Sterility test of *Ghrita Manda*.

The microbial profiling of after *Ghrita Manda* ten months of its shelf life, exhibited the presence few gram-positive cocci in clusters, pairs and occasionally in short chains and the sample did not show any the growth of Coliforms, molds, and yeasts throughout the storage period. (Shown in Fig. 6).

## 4 Discussion

Despite the benefits, chronic usage of BAC preserved eye drops results in conjunctival and corneal epithelial damages, upregulation of inflammatory markers thus precipitating dry eye symptoms. It resembles the characteristic feature of an evaporative dry eye and this vicious cycle of continues with the instillation of BAC preserved artificial tears which are usually prescribed for minimum of four times a day to maintain stable tear film volume. Murine models for DE are most commonly employed owing to their widespread availability the reagents for the immunological studies and minimal challenges in housing and its maintenance [32,33]. Also, in a study on standardizing rodent models for DE, it was found that BAC induced corneal and conjunctival changes within 5 days of its instillation and the recovery after its discontinuation of the agent showed persistence of symptoms in the rodent thus making it suitable model for DE related drug studies [34]. IL-6 levels are markedly raised in the mouse's regenerating corneal epithelium during the early stages of healing from topical instillation of BAC, suggesting that IL-6 is an essential cytokine involved in the sterile inflammation of the cornea [35]. After BAC withdrawal, DE persisted for 1-2 weeks in the 0.1% and 0.15% models and for 2 to 3 weeks in the 0.2% BAC model [36].

The findings of the experiment shows that the topical instillation of *Ghrita Manda* improved the TFBUT and fluorescein score values significantly when compared to the negative control group (p < 0.001). The histopathological analysis with H&E stain implies that *Ghrita Manda* reversed the Dry Eye pathology and maintained smooth corneal epithelial integrity when compared to negative control group. In the present study, the Control, Trial, Positive Control, and Negative Control groups exhibited a gradual increase in IL-6 expression. Elevated IL-6 expression in the BAC-only group indicated corneal inflammation and its role in promoting angiogenesis, correlates with neovascularization observed in the histology of cornea in negative control group [37]. Although the reversal of dry eye occurs once the topical instillation of BAC has been stopped, studies show that IL-6 levels may continue to be increased even after the acute remission of the disease and has been shown that persistent, dysregulated IL-6 production contributes pathologically to a number chronic inflammatory disorders of the ocular surface [38]. Notably, the Trial group showed lower IL-6 expression with 3.63 ± 0.023 than the Positive Control group 3.85 ± 1.56, suggesting that *Ghrita Manda* effectively mitigates inflammatory responses in BAC-induced dry eye. The Western blot results, with a sample size of n = 2, exhibited consistent expression patterns across duplicates, offering initial biological insights and serves as a foundation for future investigations with higher sample sizes. After five months of storage, *Ghrita Manda* exhibited peroxide and acid values of 0.39 and 0.93, respectively. In contrast, studies show that freshly prepared ghee typically has initial peroxide and acid values of around 1.64 and 0.50, which gradually increase to 8.92 and 4.56 over time due to lipid oxidation and rancidity. The reduced values in the present study drug indicate its stability during storage [39].

Microbial analysis of the *Ghrita Manda* sample revealed a total bacterial count of 4 × 10^2^ CFU/ml, indicating only a trace presence of viable microorganisms. This value falls well within the FSSAI permissible limit of 5 × 10^3^ CFU/g, confirming the product's stability and hygienic quality [40]. The ghee prepared by the indigenous (desi) method naturally retains traces of lactic acid bacteria originating from fermented dairy products used during butter preparation [41]. Interestingly, a study by Alfonso et al. reported that a one-month intervention with probiotic eye drops containing *Lactobacillus* species improved symptoms in vernal keratoconjunctivitis (VKC) without any adverse ocular effects. Furthermore, the present ghee sample showed no growth of yeast or mold, supporting its microbiological safety.

GC-MS analysis of *Ghrita Manda* exhibited the presence of essential fatty acids like linoleic acid, palmitic acid, oleic acid and glycidyl esters. Studies suggest that the oral supplementation of linoleic acid was shown to improve dry eye symptoms in human trials [42] and topical application of alpha linoleic acid significantly reduced fluorescein corneal staining in a mouse model of dry eye, and this was correlated with a reduction in the expression of IL-6 on the surface of the eye [43]. Evidence suggests that oleic and linoleic acid improves the lipid layer's distribution across the ocular surface thus making the tear film more elastic and compressible [44]. n-hexadecanoic acid is found to inhibit the action of phospholipase A_2_ which plays a key role in the ocular surface inflammation thereby producing an anti-inflammatory effect [45]. Glycidyl palmitoleate has anti-inflammatory, wound healing activity. In a stress-induced mouse dry eye model, oral administration of Palmitoleic Acid (Glycidyl palmitoleate) significantly suppressed the expression of lacrimal gland cytokines, such as eotaxin-1 and interleukin-6 and exhibited the ability to reduce inflammation of the lacrimal gland, therefore protecting the tear production capacity in dry eye disorders [46]. Additionally, ghee forms a rich source of fat-soluble vitamins (vitamin A & E) and β-carotene which is found to exhibit anti-inflammatory properties by reducing the tumor necrosis factor-α and interleukin-6 levels [47]. The outer layer of the tear film lipid layer (TFLL), also known as the air-water interface, is mainly made up of lipids that keep the ocular surface from drying up and from evaporating water while also giving the cornea a smooth visual surface. In addition to being crucial for tear surface tension, the lipid, wax esters and protein components of the TFLL are also necessary for ocular homeostasis and the physiological hydration of the ocular surface. Hence, *Ghrita Manda* helps in replenishing the TFLL and can be an ideal alternative to lubricants.

In Ayurveda, the pathogenesis of preservative induced dry eye can be considered *vata pitta dushti* in the *netra mandala* (layers of eye) resulting in the *sushkata* (dryness) of the structures. Thus, *sushkata* (dryness) of the structures initiate the *paka* (inflammation) to establish in the ocular surface. The treatment of *sushkakshipaka* of involves primarily ocular therapies with *Ghrita* based formulations. Hence, *Ghrita Maṇḍa* has been chosen as a therapeutic agent. Additionally, it retains its liquidity at room temperature and so it can be employed in the form of eye drop. Also, according to Drugs and Cosmetics (5th Amendment) Rules, 2016, rule 161-B, the shelf life of *Ghrita* is 2 years and the usage of preservatives are not employed in Ayurvedic *Ghrita* based formulations. *Netra kriyakalpa* (ocular therapeutic procedures) are the group of specialized local therapies which are employed in ocular pathologies. Among them, *Ascoyotana* (eye drop) is the foremost and finds its utilization even in the emergency conditions of the eye. The property of *Ghrita Manda* is explained as *Ghritavat*, meaning which are similar to *ghee* [48]. Owing to its *madhura rasa*, it is *Vata pittahara* in nature, thus the *dosha dushti* involved might be rectified with the topical instillation of *Ghrita Manda*. The *cakshushya* (drugs or intervention good for eye and eye sight) property of *Ghrita Manda* makes it an ideal drug of choice in the ocular pathologies. Also, with its *vishahara* (anti-toxic) nature it might have aided in decreasing the toxic effect of BAC on the ocular surface there by ameliorating the inflammation. The physical properties of *Ghrita Manda* are stated as *tanu* (thin)*, sara* (mobile), *drava* (fluidity) thus making it apt for using it for *Ascoyotana* (eye drop).

## 5 Conclusion

In the recent trend of dry eye treatment, the usage of Essential fatty acids is widely recommended owing to the lubricative, ocular surface epithelial protection and anti-inflammatory properties of the lipid particles. GC-MS analysis of *Ghrita Manda* demonstrated the presence of Essential Fatty Acids and esters. The present study demonstrated the enhanced tear film stability and maintained the corneal epithelial integrity and ameliorated the IL-6 expression in the cornea which fulfils the ideal dry eye treatment involving lubrication, corneal epithelial protection and anti-inflammatory action thereby have a potential effect in alleviating the dry eye symptoms.

## Sources of funding

We have not received any funding for this reporting.

## Author contributions

RSJ: Conceptualization, Methodology/ Study design, Validation, Formal analysis, Investigation, Resources, Data curation, Writing – original draft, Writing – review and editing, Visualization, Project administration; SK: Conceptualization, Methodology/ Study design, Validation, Formal analysis, Writing – original draft, Writing – review and editing, Visualization, Supervision; ABN: Conceptualization, Methodology/ Study design, Validation, Formal analysis, Data curation, Writing – original draft, Writing – review and editing, Visualization, Supervision, Project administration.

## Declaration of generative AI in scientific Writing

During the preparation of this work, the author(s) used ChatGPT in order to improve the readability and language of the manuscript. After using this tool/service, the author(s) reviewed and edited the content as needed and take(s) full responsibility for the content of the publication.

## Declaration of competing interest

The authors declare that they have no known competing financial interests or personal relationships that could have appeared to influence the work reported in this paper.
